# Physical activity may not be associated with long‐term risk of dementia and Alzheimer’s disease

**DOI:** 10.1111/eci.13415

**Published:** 2020-10-14

**Authors:** Setor K. Kunutsor, Jari A. Laukkanen, Jussi Kauhanen, Peter Willeit

**Affiliations:** ^1^ National Institute for Health Research Bristol Biomedical Research Centre University Hospitals Bristol and Weston NHS Foundation Trust and the University of Bristol Bristol UK; ^2^ Musculoskeletal Research Unit Translational Health Sciences Bristol Medical School Learning & Research Building (Level 1) Southmead Hospital University of Bristol Bristol UK; ^3^ Institute of Public Health and Clinical Nutrition University of Eastern Finland Kuopio Finland; ^4^ Faculty of Sport and Health Sciences University of Jyväskylä Jyväskylä Finland; ^5^ Central Finland Health Care District Hospital District Jyväskylä Finland; ^6^ Department of Neurology Medical University of Innsbruck Innsbruck Austria; ^7^ Department of Public Health and Primary Care University of Cambridge Cambridge UK

**Keywords:** Alzheimer's disease, cohort study, dementia, physical activity, risk factor

## Abstract

**Background:**

While it is well established that physical activity is associated with reduced risk of vascular and nonvascular outcomes as well as mortality, evidence on the association between physical activity and dementia is inconsistent. We aimed to assess the associations of physical activity with the risk of dementia and Alzheimer's disease (AD).

**Material and methods:**

We analysed data on 2394 apparently healthy men with good baseline cognitive function from the prospective population‐based Kuopio Ischaemic Heart Disease study. We assessed habits of physical activity at baseline using a 12‐month leisure time physical activity (LTPA) questionnaire. Using Cox regression, we calculated hazard ratios adjusted for body mass index, systolic blood pressure, smoking status, history of type‐2 diabetes, total cholesterol, high‐density lipoprotein cholesterol, alcohol consumption, history of coronary heart disease and high‐sensitivity C‐reactive protein.

**Results:**

During a median follow‐up of 24.9 years (interquartile range: 18.3‐26.9), 208 men developed dementia and 128 developed AD. Multivariable adjusted hazard ratios for dementia comparing top vs bottom tertiles of physical activity were 0.97 (95% confidence intervals: 0.69‐1.38) for total physical activity volume, 0.96 (0.69‐1.34) for conditioning LTPA volume and 1.13 (0.80‐1.61) for total LTPA volume. Corresponding hazard ratios for AD were 1.19 (0.76‐1.85), 0.98 (0.64‐1.49) and 1.22 (0.77‐1.93). Associations were consistent in analyses restricted to participants with ≥10 years of follow‐up.

**Conclusions:**

In middle‐aged Caucasian men, various physical activity exposures were not associated with all‐cause dementia or AD. Future studies should address biases due to reverse causation and regression dilution and should involve objective measures of physical activity.

## 1 INTRODUCTION

The role of physical activity in disease prevention has been studied widely and is well established. Physical activity is associated with reduced risk of vascular and nonvascular outcomes as well as mortality.^1^, ^2^ This link is likely to be mediated through several physiological and metabolic processes, including improved vascular risk markers such as body weight, blood pressure, natriuretic peptides, and lipid profiles,^3^, ^4^, ^5^ anti‐inflammatory effects,^6^ reduced oxidative stress and enhanced cardiovascular function.^7^ Furthermore, vigorous or high‐intensity physical activity may be associated with substantially greater benefits compared with physical activity of low or moderate intensity.^8^

Dementia—a neurodegenerative disorder characterised by progressive deterioration of cognitive functions—represents a growing public health burden and is one of the major challenges of the century. Alzheimer's disease (AD) is the most common cause of dementia; vascular dementia is the second most common cause. Global prevalence of AD is projected to increase from 30 million in 2010 to 106 million in 2050.^9^ It is estimated that one‐third of AD cases may be attributable to modifiable risk factors^10^ (e.g. type‐2 diabetes, hypertension, obesity, dyslipidaemia, smoking, smoking, depression) and non‐modifiable risk factors^11^ (e.g. ageing, the *APOE* polymorphism). Still, its pathogenesis is still not fully understood and it appears that other potential risk factors may be involved in AD development.

Several prospective cohorts have investigated the associations of physical activity with the risk of dementia or AD, but they yielded inconsistent results.^12^, ^13^, ^14^, ^15^ Whereas some studies reported inverse relationships (i.e. a decreased dementia risk with higher physical activity),^13^, ^14^ others found no evidence of association.^12^, ^15^ To help clarify these associations, we set out to assess the relationships of physical activity with the long‐term risk of all‐cause dementia and AD in the Finnish Kuopio Ischaemic Heart Disease (KIHD) population‐based prospective cohort study. Several different physical activity exposures are available within the KIHD study database, enabling us to evaluate the nature and magnitude of the associations in greater detail than in previous studies.

## 2 METHODS

### 2.1 Study design and participants

Reporting of the study conforms to broad EQUATOR guidelines (Table S1).^16^

**Figure 1:**
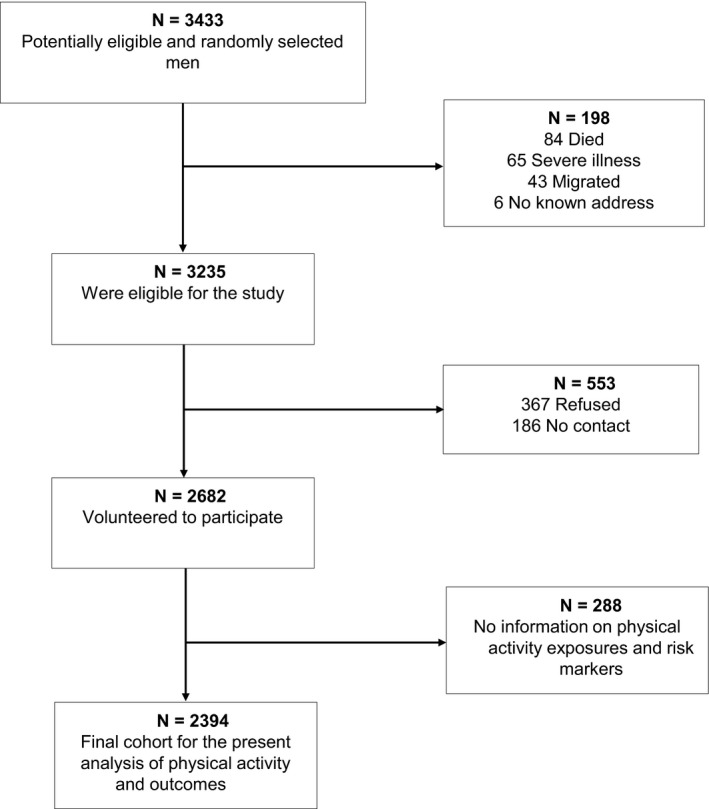
Derivation of the analytic sample

The analyses used data from the KIHD study, a population‐based prospective cohort conducted in eastern Finland. Details on participant recruitment have been described previously.^17^ In brief, a representative sample of 3433 men aged 42‐61 years were randomly selected from the city of Kuopio and its neighbouring rural communities. Of the 3433 randomly selected men, 198 were excluded because of death (n = 84), serious disease (n = 65) or migration (n = 49), leaving 3235 eligible to participate in the study. Of the 3235 eligible men, 2682 volunteered to participate, 186 did not respond to the invitation, and 367 declined to give informed consent. Baseline examinations were conducted between March 1984 and December 1989. The current study was based on 2394 men with complete baseline data on physical activity exposures, relevant covariates and dementia outcomes. The derivation of the analytic sample is described in Figure 1. All study procedures were approved by the local ethical committee and conducted according to the Declaration of Helsinki. All participants provided written informed consent.

### 2.2 Ascertainment of outcomes

We included all events of all‐cause dementia and AD cases that occurred from study entry through 31 December 2013. There were no losses to follow‐up because all study participants are under continuous annual monitoring (using personal identification codes) for incident outcomes, including dementia and AD. Data on outcomes were ascertained from record linkage to the national computerised hospitalisation registry covering every hospitalisation in Finland, comprehensive review of hospital admission and discharge records, medico‐legal reports and inpatient physician claims data. At baseline and every subsequent year, patients underwent cognition tests including the Mini‐Mental State Examination and Geriatric Mental State test.^18^ Participants that were deemed positive in this screening then underwent further testing, including examinations by neurologists, neuropsychological testing and magnetic resonance imaging of the brain. An independent committee of physicians reviewed all potential cases of dementia to obtain a consensus on the diagnosis and aetiology.^19^

### 2.3 Measurement of covariates

All assessments of covariates including blood biomarkers, physical measurements and physical activity exposures were conducted at baseline study entry. Details on methodologies for collection and biochemical analysis of blood specimen have been previously described.^17^ Briefly, before assessment, participants fasted overnight before blood collection and abstained from alcohol consumption for ≥3 days and from smoking for ≥12 hours. Serum samples were stored at −80°C before analysis. Serum high‐sensitivity C‐reactive protein (hsCRP) was measured with the Immulite High Sensitivity C‐Reactive Protein Assay (DPC, Los Angeles, CA, USA). Resting blood pressure was measured between 8:00 and 10:00 am with a random‐zero sphygmomanometer. Alcohol consumption was assessed using the Nordic Alcohol Consumption Inventory. Age, smoking, medical history and physical activity were assessed with a self‐administered health and lifestyle questionnaire.

### 2.4 Measurement of physical activity exposures

Habits of leisure time physical activities were assessed by a trained interviewer using the KIHD 12‐month leisure time physical activity (LTPA) questionnaire.^20^ The questionnaire is quantitative and was modified from the Minnesota LTPA Questionnaire.^21^ It captures common leisure time physical activities in Finland (conditioning activities, e.g. walking, skiing, bicycling, swimming, rowing, ball games; non‐conditioning activities, e.g. repairs, crafts, gardening, building, hunting, fishing) and assesses these activities over the previous year. For each type of physical activity, frequency (sessions per month), average duration (hours and minutes per session) and intensity (scored as 0 for recreational activity, 1 for conditioning activity, 2 for brisk conditioning activity and 3 for competitive, strenuous exercise) were recorded. Based on the energy cost of each physical activity type, a metabolic equivalent task (MET, or metabolic equivalents of oxygen consumption) score was assigned to its intensity. One metabolic unit corresponds to an energy expenditure of approximately 1 kcal per kilogram of body weight per hour.^22^ As previously reported, the KIHD LTPA Questionnaire showed high reproducibility and provides a useful measure of average weekly leisure time activity over a 1‐year period.^23^

Occupational physical activity (OPA) was assessed using a self‐reported questionnaire in men who had worked—full‐time or part‐time—in the past 12 months.^24^ The questionnaire assessed the durations of sitting, standing, walking on level ground, walking on uneven ground, climbing stairs and other activities during a typical working day. In addition, for participants currently in employment, it assessed workdays per week, the number of hours and minutes worked per day, and the number of days they missed work due to illness during the past 12 months. For participants currently not in employment, it assessed the year when an unemployment or retirement period began, the number of days worked per week in the last job and number of hours worked per day. The 12‐month test‐retest correlations for OPA was 0.69, indicating good reliability of the instrument.^23^

Total physical activity included both leisure time and OPA and was classified into conditioning vs non‐conditioning components. Total physical activity volume (MET h/y) was estimated by multiplying the intensity in METs by the duration. The present analysis used the measures of total physical activity volume, conditioning LTPA volume, and total LTPA volume.

### 2.5 Statistical analyses

Baseline participant characteristics were summarised using descriptive analyses. Cross‐sectional correlates of total physical activity volume were assessed using age‐adjusted Pearson correlation coefficients. Cox regression was used to assess associations of tertiles of physical activity exposures at baseline with incident outcomes. The assumption of proportional hazards was tested using Schoenfeld residuals and was met.^25^ Hazard ratios (HRs) were adjusted for age (Model 1) or for age, body mass index (BMI), baseline systolic blood pressure (SBP), smoking status, history of type‐2 diabetes, total cholesterol, high‐density lipoprotein cholesterol (HDL‐C), alcohol consumption, history of coronary heart disease (CHD) and hsCRP (Model 2). To assess the potential impact of reverse causation bias, we conducted sensitivity analyses that limited analyses to participants with at least 10 years of follow‐up. The 10‐year threshold was chosen for consistency with a previous report^12^ and also because it has been reported that physical activity tends to decline approximately a decade before dementia diagnosis.^15^ Other sensitivity analyses excluded participants with pre‐existing CHD. Analyses were conducted with Stata version 15.1 (Stata Corp, College Station, Texas).

## 3 RESULTS

### 3.1 Baseline characteristics

Table 1 summarises baseline characteristics of the 2394 participants included in the analysis. Mean age was 53 years (standard deviation 5), mean total physical activity volume was 17 969 MET h/y (standard deviation 3707), and median volumes of conditioning and total LTPA were 412 (interquartile range 169‐890) and 1393 (774‐2306) MET h/y, respectively. In a cross‐sectional analysis, there were weak inverse correlations of total physical activity volume with alcohol consumption, BMI, fasting plasma glucose and hsCRP, and weak positive correlations with total LTPA and HDL‐C (Table 1).

**Table 1:** Baseline participant characteristics

	Overall (N = 2394) Mean (SD) or median (IQR) or n (%)	Pearson correlation *r* (95% CI)^a^
Total PA volume (MET h/y)	17 969 (3707)	‐
***Questionnaire/Prevalent conditions***		
Age at survey (y)	53 (5)	−0.04 (−0.08, 0.00)
Alcohol consumption (g/wk)	32.0 (6.4‐90.1)	−0.09 (−0.13, −0.05)***
History of diabetes	125 (5.2)	‐
Current smokers	719 (30.0)	‐
History of CHD	597 (24.9)	‐
***Physical measurements***		
BMI (kg/m^2^)	26.9 (3.5)	−0.08 (−0.12, −0.04)***
SBP (mm Hg)	134 (17)	−0.00 (−0.04, 0.04)
DBP (mm Hg)	89 (10)	−0.03 (−0.07, 0.01)
Conditioning LTPA volume (MET h/y)	412 (169‐890)	−0.04 (−0.08, 0.00)
Total LTPA volume (MET h/y)	1393 (774‐2306)	0.07 (0.03, 0.11)*
***Lipid markers***		
Total cholesterol (mmol/L)	5.90 (1.09)	0.03 (−0.01, 0.07)
HDL‐C (mmol/L)	1.29 (0.30)	0.16 (0.13, 0.20)***
***Metabolic markers***		
Fasting plasma glucose (mmol/L)	5.34 (1.24)	−0.07 (−0.11, −0.03)**
High‐sensitivity CRP (mg/L)	1.27 (0.70‐2.38)	−0.07 (−0.11, −0.03)**

Abbreviations: BMI, body mass index; CHD, coronary heart disease; CRP, C‐reactive protein; DBP, diastolic blood pressure; HDL‐C, high‐density lipoprotein cholesterol; IQR, interquartile range; LTPA, leisure time physical activity; SBP, systolic blood pressure; SD, standard deviation; TPA, total physical activity.

^a^ age‐adjusted Pearson correlation coefficients between TPA volume and the row variables; asterisks indicate the level of statistical significance:

* *P* < .05;

** *P* < .01;

*** *P* < .001.

### 3.2 Physical activity and risk of dementia and AD

During a median follow‐up of 24.9 (interquartile range, 18.3‐26.9) years, we recorded 208 cases of incident dementia and 128 cases of incident AD. This corresponded to annual incidence rates of 3.98 (95% CI: 3.48‐4.56) and 2.44 (2.05‐2.90) per 1000 person‐years at risk.

For dementia, age‐adjusted HRs comparing the top versus the bottom tertile group were 1.02 (95% CI: 0.73‐1.43) for total physical activity volume, 0.93 (0.67‐1.29) for conditioning LTPA volume and 1.13 (0.80‐1.60) for total LTPA (Table 2). Associations remained virtually unchanged upon multivariable adjustment for age, BMI, SBP, smoking status, history of type‐2 diabetes, total cholesterol, HDL‐C, alcohol consumption, history of CHD and hsCRP, with HRs of 0.97 (0.69‐1.38), 0.96 (0.69‐1.34) and 1.13 (0.80‐1.61), respectively.

**Table 2:** Associations of physical activity exposures with risk of all‐cause dementia

Physical activity exposure	Events/Total	Model 1		Model 2	
		HR (95% CI)	*P‐*value	HR (95% CI)	*P‐*value
**TPA volume (MET h/y)**					
Tertile 1 (9350‐15 907)	64/804	ref		ref	
Tertile 2 (15 908‐18 810)	72/794	0.99 (0.70‐1.38)	.94	0.98 (0.70‐1.38)	.92
Tertile 3 (18 811‐35 703)	72/796	1.02 (0.73‐1.43)	.91	0.97 (0.69‐1.38)	.88
**Conditioning LTPA volume (MET h/y)**					
Tertile 1 (0.53‐239.42)	73/798	ref		ref	
Tertile 2 (239.43‐677.64)	62/798	0.81 (0.58‐1.14)	.24	0.86 (0.61‐1.20)	.37
Tertile 3 (677.65‐8981.03)	73/798	0.93 (0.67‐1.29)	.66	0.96 (0.69‐1.34)	.82
**Total LTPA volume (MET h/y)**					
Tertile 1 (5.46‐981.07)	57/798	ref		ref	
Tertile 2 (981.08‐1972.84)	74/798	1.22 (0.87‐1.73)	.25	1.24 (0.88‐1.76)	.23
Tertile 3 (1972.85‐22 621.86)	77/798	1.13 (0.80‐1.60)	.47	1.13 (0.80‐1.61)	.48

Abbreviations: CI, confidence interval; HR, hazard ratio; ref, reference; TPA, total physical activity.

Model 1: Adjusted for age.

Model 2: Model 1 plus body mass index, baseline systolic blood pressure, smoking status, history of type 2 diabetes, total cholesterol, high‐density lipoprotein cholesterol, alcohol consumption, history of coronary heart disease and CRP.

For AD, age‐adjusted HRs were 1.19 (95% CI: 0.77‐1.84) for total physical activity volume, 0.91 (0.60‐1.38) for conditioning LTPA volume and 1.19 (0.76‐1.87) for total LTPA (Table 3). Again, HRs for AD were comparable when multivariable adjustment was employed (1.19 [0.76‐1.85], 0.98 [0.64‐1.49] and 1.22 [0.77‐1.93]).

**Table 3:** Associations of physical activity exposures with risk of Alzheimer's disease

Physical activity exposure	Events/Total	Model 1		Model 2	
		HR (95% CI)	*P‐*value	HR (95% CI)	*P‐*value
**TPA volume (MET h/y)**					
Tertile 1 (9350‐15 907)	36/804	ref		ref	
Tertile 2 (15 908‐18 810)	44/794	1.06 (0.68‐1.64)	.81	1.06 (0.68‐1.66)	.79
Tertile 3 (18 811‐35 703)	48/796	1.19 (0.77‐1.84)	.42	1.19 (0.76‐1.85)	.45
**Conditioning LTPA volume (MET h/y)**					
Tertile 1 (0.53‐239.42)	45/798	ref		ref	
Tertile 2 (239.43‐677.64)	39/798	0.84 (0.55‐1.29)	.43	0.90 (0.58‐1.39)	.64
Tertile 3 (677.65‐8981.03)	44/798	0.91 (0.60‐1.38)	.65	0.98 (0.64‐1.49)	.91
**Total LTPA volume (MET h/y)**					
Tertile 1 (5.46‐981.07)	32/798	ref		ref	
Tertile 2 (981.08‐1972.84)	50/798	1.48 (0.95‐2.30)	.09	1.53 (0.98‐2.39)	.06
Tertile 3 (1972.85‐22 621.86)	46/798	1.19 (0.76‐1.87)	.45	1.22 (0.77‐1.93)	.39

Abbreviations: CI, confidence interval; HR, hazard ratio; ref, reference; TPA, total physical activity.

Model 1: Adjusted for age.

Model 2: Model 1 plus body mass index, baseline systolic blood pressure, smoking status, history of type 2 diabetes, total cholesterol, high‐density lipoprotein cholesterol, alcohol consumption, history of coronary heart disease and CRP.

In sensitivity analyses, results were consistent in: (a) analyses limited to participants with at least 10 years of follow‐up (Table S2) and (b) analyses excluding men with a history of CHD (data not shown).

## 4 DISCUSSION

In this Finnish population‐based prospective study comprised of middle‐aged Caucasian men with good cognitive function at baseline, there were no associations between physical activity and risk of developing dementia or AD over a follow‐up of 24.9 years. Results were robust in analyses restricted to participants with at least 10 years of follow‐up and analysis excluding participants with prior CHD. The overall incidence rate of dementia was 3.98/1000 person‐years, which is in line with the latest estimates reported by the World Health Organization.^26^

Prior individual studies and aggregate reviews have reported divergent findings on the associations between physical activity and dementia risk. In consistence with our study, several prior studies reported null associations. The Honolulu‐Asia Aging Study involving 3468 middle‐aged Japanese American men found no association between physical activity and AD diagnosed 25 years following cohort entry.^27^ The Whitehall II cohort study found no association of physical activity with 27‐year dementia risk.^15^ As the largest published study so far, Kivimäki and colleagues showed that physical inactivity was not associated with all‐cause dementia or AD in an individual participant data (IPD) meta‐analysis of 19 prospective studies with approximately 400 000 participants in total.^12^ In contrast to these and our findings, there are also several other studies that reported inverse associations between physical activity and the risk of dementia.^13^, ^14^, ^28^, ^29^, ^30^, ^31^, ^32^ For example, in a cohort of 4406 persons aged 61‐97 years with a follow‐up period of up to 14 years, higher physical activity was associated with a lower risk of dementia, but only in the initial 4 years of follow‐up.^31^ In a study in 3547 participants aged ≥55 years followed up for 2.6 years, dementia risk was higher in participants with moderate or poor levels of physical activity.^30^ In 15 589 community‐living Chinese aged ≥65 years without baseline dementia, participation in leisure physical activities was associated with decreased risk of dementia over an average follow‐up of 6 years.^32^

Given the well‐established link between physical activity and several chronic disease outcomes and the notion that dementia and some of these outcomes such as CVD, diabetes and cancer share common risk factors, the current findings may seem unexpected. Even though pathways such as inflammation and oxidative stress are implicated in the pathogenesis of these outcomes as well as dementia,^33^, ^34^ null associations in epidemiological studies point towards important differences in how physical activity affects the pathogenesis of these different diseases.

Specific study design feature and population factors may explain the heterogeneous findings in prior studies. First, studies may have been affected by reverse causation bias, as physical activity tends to decline in the early phases of dementia before clinical diagnosis.^15^ Consistent with this hypothesis, significant findings have been demonstrated predominantly in studies which short‐term follow‐up durations^30^, ^31^, ^32^ and null findings in long‐term follow‐up studies.^12^, ^15^, ^27^ In our current study, there was no evidence of associations over the entire follow‐up duration of 25 years. In line, the large‐scale IPD meta‐analysis mentioned above^12^ reported associations with all‐cause dementia and AD only when physical activity was assessed <10 years before diagnosis and not when it was assessed ≥10 years before. Several meta‐analyses demonstrating inverse associations between physical activity and dementia risk have predominantly included studies with follow‐up durations of <10 years.^13^, ^28^, ^29^

Second, one also has to consider differences between study population such as age, sex or genetic background. Many of the prior studies were based in elderly populations,^31^, ^35^, ^36^, ^37^ hence increasing the likelihood of reverse causation bias contributing to the beneficial impact of physical activity on dementia risk. Whether sex could be an effect modifier of the association between physical activity and dementia risk is still unclear. Two prior studies have shown significant association only in women and not in men,^38^, ^39^ but the aforementioned IPD meta‐analysis did not confirm this observation.^12^

Third, the null association demonstrated in these studies on dementia and AD could also be due to lack of objective assessments of physical activity and the phenomenon of regression dilution bias^40^ as a result of employing single baseline measurements of physical activity. Taking all the data together, there is insufficient evidence to suggest that physical activity could prevent dementia or AD.

Our study has several strengths, including a prospective cohort design, a large sample size, a sample representative of the general population, a high participation rate and completeness of follow‐up information. Mean follow‐up duration in our study was sufficiently long to ascertain risk for dementia. The relatively healthy cohort of middle‐aged individuals with good cognitive function limited the possibilities of reverse causation bias. Our study included several different physical activity exposures and was able to adjust for relevant confounders. A limitation of our study is that physical activity was self‐reported, hence allowing some misclassification or reporting bias. For OPA, given that lifetime job stability among people living in the Kuopio region is high,^41^ the scope of misclassification is expected to be low. Furthermore, we assessed physical activity at a single time point and could therefore not correct for regression dilution bias,^40^ which potentially results in the underestimation of the true association between an exposure and outcome, particularly for cohort studies with long‐term follow‐up. However, data from the KIHD study suggest that the 12‐month intra‐individual variability in physical activity exposure is relatively small and hence recommended for population studies of this nature.^23^ Due to the low incidence rates of dementia and AD and hence few events in the first 10 years of follow‐up, we were unable to conduct sufficiently powered targeted analyses of short‐term dementia risk. Furthermore, the findings were based on middle‐aged Caucasian men and therefore cannot be generalised to women, other age groups and populations. Finally, the analyses focused on all‐cause dementia and AD only, given the unavailability of specific dementia subtypes such as vascular dementia.

In conclusion, in analyses based on approximately healthy middle‐aged Caucasian men, different physical activity exposures were not associated with all‐cause dementia or AD. Previous findings of a protective association could be attributed to reverse causation. Future studies should address biases due to reverse causation and regression dilution and should involve objective measures of physical activity.

## CONFLICT OF INTEREST

No potential conflict of interest was reported by the authors.

## AUTHOR CONTRIBUTIONS

SKK, JAL and PW designed the study, analysed data and wrote the manuscript. JAL and JK collected data. JK revised the manuscript for intellectual content. PW is the guarantor of this work and, as such, had full access to all the data in the study and takes responsibility for the integrity of the data and the accuracy of the data analysis.

## Supporting information

Table S1‐S2
