# Supplementary material for: Physical activity may not be associated with long‐term risk of dementia and Alzheimer’s disease
Source: Eur J Clin Invest. 2020 Oct 14;51(3):e13415. doi: 10.1111/eci.13415 (PMC7988584; doi:10.1111/eci.13415)
Supplement: Supplementary file 1 — Table S1‐S2 [file ECI-51-e13415-s001.pdf]

Supplementary Material

**Table S1**	STROBE statement
**Table S2**	Association of physical activity exposures with dementia and Alzheimer’s disease, in the principal analysis compared to the sensitivity analysis restricted to $\geq$ 10 years follow-up

Table S1. STROBE 2007 Statement—Checklist of items that should be included in reports of cohort studies

Section/Topic	Item #	Recommendation	Reported on page #
**Title and abstract**	1	(*a*) Indicate the study's design with a commonly used term in the title or the abstract	Page 1
		(*b*) Provide in the abstract an informative and balanced summary of what was done and what was found	Page 2
**Introduction**			
Background/rationale	2	Explain the scientific background and rationale for the investigation being reported	Introduction
Objectives	3	State specific objectives, including any prespecified hypotheses	Introduction
**Methods**			
Study design	4	Present key elements of study design early in the paper	Study Design and Participants
Setting	5	Describe the setting, locations, and relevant dates, including periods of recruitment, exposure, follow-up, and data collection	Study Design and Participants
Participants	6	(*a*) Give the eligibility criteria, and the sources and methods of selection of participants. Describe methods of follow-up	Study Design and Participants
		(*b*) For matched studies, give matching criteria and number of exposed and unexposed	Not applicable
Variables	7	Clearly define all outcomes, exposures, predictors, potential confounders, and effect modifiers. Give diagnostic criteria, if applicable	Measurement of covariates and physical activity exposures
Data sources/ measurement	8*	For each variable of interest, give sources of data and details of methods of assessment (measurement). Describe comparability of assessment methods if there is more than one group	Measurement of covariates and physical activity exposures
Bias	9	Describe any efforts to address potential sources of bias	Statistical Analyses
Study size	10	Explain how the study size was arrived at	Statistical Analyses
Quantitative variables	11	Explain how quantitative variables were handled in the analyses. If applicable, describe which groupings were chosen and why	Statistical Analyses
Statistical methods	12	(*a*) Describe all statistical methods, including those used to control for confounding	Statistical Analyses
		(*b*) Describe any methods used to examine subgroups and interactions	Statistical Analyses
		(*c*) Explain how missing data were addressed	Not applicable
		(*d*) If applicable, explain how loss to follow-up was addressed	Not applicable
		(*e*) Describe any sensitivity analyses	Statistical Analyses

**Results**			
Participants	13*	(a) Report numbers of individuals at each stage of study—eg numbers potentially eligible, examined for eligibility, confirmed eligible, included in the study, completing follow-up, and analysed	Study Design and Participants
		(b) Give reasons for non-participation at each stage	Study Design and Participants
		(c) Consider use of a flow diagram	
Descriptive data	14*	(a) Give characteristics of study participants (eg demographic, clinical, social) and information on exposures and potential confounders	Results; Tables 1
		(b) Indicate number of participants with missing data for each variable of interest	
		(c) Summarise follow-up time (eg, average and total amount)	Results
Outcome data	15*	Report numbers of outcome events or summary measures over time	Results
Main results	16	(*a*) Give unadjusted estimates and, if applicable, confounder-adjusted estimates and their precision (eg, 95% confidence interval). Make clear which confounders were adjusted for and why they were included	Results; Figures 2-3
		(*b*) Report category boundaries when continuous variables were categorized	Results; Figures 2-3
		(*c*) If relevant, consider translating estimates of relative risk into absolute risk for a meaningful time period	
Other analyses	17	Report other analyses done—eg analyses of subgroups and interactions, and sensitivity analyses	Results; Appendix 2
**Discussion**			
Key results	18	Summarise key results with reference to study objectives	Discussion
**Limitations**			
Interpretation	20	Give a cautious overall interpretation of results considering objectives, limitations, multiplicity of analyses, results from similar studies, and other relevant evidence	Discussion
Generalisability	21	Discuss the generalisability (external validity) of the study results	Discussion
**Other information**			
Funding	22	Give the source of funding and the role of the funders for the present study and, if applicable, for the original study on which the present article is based	Page 14

Table S2. Association of physical activity exposures with dementia and Alzheimer's disease, in the principal analysis compared to the sensitivity analysis restricted to ≥ 10 years follow-up

	Principal analysis			Analysis restricted to participants with at least 10 years of follow-up		
	No. of events / participants	HR (95% CI), multivariable adjusted*	*P*-value	No. of events / participants	HR (95% CI), multivariable adjusted*	*P*-value
**Dementia**						
Total PA						
Tertile 1	64 / 804	[Reference]		62 / 714	[Reference]	
Tertile 2	72 / 794	0.98 (0.70, 1.38)	0.92	70 / 711	1.03 (0.73, 1.45)	0.86
Tertile 3	72 / 796	0.97 (0.69, 1.38)	0.88	69 / 710	0.99 (0.70, 1.42)	0.97
Conditioning LTPA						
Tertile 1	73 / 798	[Reference]		71 / 712	[Reference]	
Tertile 2	62 / 798	0.86 (0.61, 1.20)	0.37	60 / 712	0.84 (0.59, 1.19)	0.32
Tertile 3	73 / 798	0.96 (0.69, 1.34)	0.82	70 / 711	0.93 (0.67, 1.31)	0.70
Total LTPA						
Tertile 1	57 / 798	[Reference]		54 / 712	[Reference]	
Tertile 2	74 / 798	1.24 (0.88, 1.76)	0.23	73 / 712	1.31 (0.92, 1.87)	0.13
Tertile 3	77 / 798	1.13 (0.80, 1.61)	0.48	74 / 711	1.12 (0.79, 1.60)	0.53
**Alzheimer's disease**						
Total PA						
Tertile 1	36 / 804	[Reference]		36 / 716	[Reference]	
Tertile 2	44 / 794	1.06 (0.68, 1.66)	0.79	43 / 712	1.07 (0.69, 1.68)	0.76
Tertile 3	48 / 796	1.19 (0.76, 1.85)	0.45	45 / 710	1.14 (0.73, 1.80)	0.56
Conditioning LTPA						
Tertile 1	45 / 798	[Reference]		44 / 713	[Reference]	
Tertile 2	39 / 798	0.90 (0.58, 1.39)	0.64	38 / 713	0.88 (0.57, 1.37)	0.57
Tertile 3	44 / 798	0.98 (0.64, 1.49)	0.91	42 / 712	0.94 (0.61, 1.44)	0.77
Total LTPA						
Tertile 1	32 / 798	[Reference]		31 / 713	[Reference]	
Tertile 2	50 / 798	1.53 (0.98, 2.39)	0.06	49 / 713	1.57 (1.00, 2.48)	0.05
Tertile 3	46 / 798	1.22 (0.77, 1.93)	0.39	44 / 712	1.18 (0.74, 1.88)	0.49

*Adjusted for age, body-mass index, systolic blood pressure, smoking status, history of type-2 diabetes, total cholesterol, high-density lipoprotein cholesterol, alcohol consumption, history of coronary heart disease, and high-sensitivity C-reactive protein. CI, confidence interval; HR, hazard ratio; LTPA, leisure-time physical activity, PA, physical activity.
